# Characterization of *Mycobacterium orygis*

**DOI:** 10.3201/eid1903.121005

**Published:** 2013-03

**Authors:** Jakko van Ingen, Roland Brosch, Dick van Soolingen

**Affiliations:** Author affiliations: Radboud University Nijmegen Medical Center, Nijmegen, the Netherlands (J. van Ingen, D. van Soolingen);; Institut Pasteur, Paris, France (R. Brosch);; National Institute for Public Health and the Environment (RIVM), Bilthoven, the Netherlands (D. van Soolingen)

**Keywords:** tuberculosis and other mycobacteria, oryx bacillus, Mycobacterium orygis

**To the Editor:** We thank Gey van Pittius and colleagues for their addition to the markers that identify *Mycobacterium orygis* as a distinct subspecies in the *M. tuberculosis* complex (*1*). Its isolation from a wild buffalo broadens the host range of *M. orygis*. Gey van Pittius and colleagues raise 3 issues: the utility of the *gyrB*^oryx^ single-nucleotide polymorphism (SNP) being equally specific as the reported SNP in *Rv2042^38^*, the presence of genomic regions RD701 and RD702 in *M. orygis*, and the addition of the sequence type (ST) 701 spoligotype to *M. orygis*–specific spoligotypes.

We agree that use of the *gyrB*^oryx^ mutation is more practical for routine daily use because this gene helps identify several subspecies of the *M. tuberculosis* complex. However, use of the partial *Rv2042* sequencing is similarly practical because it can be combined with sequencing of the adjacent *pncA* gene, which enables identification of several *M. tuberculosis* complex species and some subspecies (i.e., *M. orygis*, *M. bovis*, *M. canettii*) (*2*), to identify the CAS genotype of *M. tuberculosis* (J. van Ingen, unpub. data) and, to some degree, assess susceptibility to pyrazinamide (*3*).

With the added data, we can conclude that *M. orygis* is an *M. tuberculosis* complex subspecies defined by the presence of genomic regions RD1, RD2, RD4, RD5a, RD6, RD13–RD16, RD701, and RD702, by the C-to-G SNP in *mmpL6*^551^, and by the deletion of regions RD3, RD5b, RD7-RD12, RDoryx_1, RDoryx_4, and RDoryx_wag22. Subspecies-specific SNPs are present in *gyrB* and *Rv2042*. Spoligotypes ST587, ST701, and closely related types are characteristic of *M. orygis*, and this subspecies yields 17–20 copies of insertion sequence *6110* and a distinct 24-locus variable number tandem repeats pattern (*4*,*5*). Given the rapid progress in genome sequencing, additional markers specific for the different subspecies will further enrich this panel of differences.
